# Pauli’s Electron in Ehrenfest and Bohm Theories, a Comparative Study

**DOI:** 10.3390/e25020190

**Published:** 2023-01-18

**Authors:** Asher Yahalom

**Affiliations:** 1 Department of Electrical & Electronic Engineering, Faculty of Engineering, Ariel University, Ariel 40700, Israel; asya@ariel.ac.il; Tel.: +972-54-7740294; 2 Center for Astrophysics, Geophysics, and Space Sciences (AGASS), Ariel University, Ariel 40700, Israel

**Keywords:** spin, quantum mechanics, Ehrenfest theorem

## Abstract

Electrons moving at slow speeds much lower than the speed of light are described by a wave function which is a solution of Pauli’s equation. This is a low-velocity limit of the relativistic Dirac equation. Here we compare two approaches, one of which is the more conservative Copenhagen’s interpretation denying a trajectory of the electron but allowing a trajectory to the electron expectation value through the Ehrenfest theorem. The said expectation value is of course calculated using a solution of Pauli’s equation. A less orthodox approach is championed by Bohm, and attributes a velocity field to the electron also derived from the Pauli wave function. It is thus interesting to compare the trajectory followed by the electron according to Bohm and its expectation value according to Ehrenfest. Both similarities and differences will be considered.

## 1. Introduction

Quantum mechanics is usually interpreted by the Copenhagen approach. This approach objects to the physical reality of the quantum wave function and declares it to be epistemological (a tool for estimating probability of measurements) in accordance with the Kantian [1] depiction of reality, and its denial of the human ability to grasp any thing in its reality (ontology). However, we also see the development of another approach of prominent scholars that think about quantum mechanics differently. This school believes in the ontological existence of the wave function. According to this approach the wave function is an element of reality much like an electromagnetic field. This was supported by Einstein and Bohm [2,3,4] has resulted in different understandings of quantum mechanics among them the fluid realization championed by Madelung [5,6] which stated that the modulus square of the wave function is a fluid density and the phase is a potential of the velocity field of the fluid.

A non-relativistic quantum equation for a spinor was first introduced by Wolfgang Pauli in 1927 [7], this was motivated by the need to explain the Stern–Gerlach experiments. Later it was shown that the Pauli equation is a low-velocity limit of the relativistic Dirac equation (see for example [8] and references therein). This equation is based on a two dimensional operator matrix Hamiltonian. Two-dimensional operator matrix Hamiltonians are common in the literature [9,10,11,12,13,14,15,16,17,18,19,20,21,22] and describe many types of quantum systems. A Bohmian analysis of the Pauli equation was given by Holland and others [3,4], however, the analogy of the Pauli theory to fluid dynamics and the notion of spin vorticity were not considered. In [23] spin fluid dynamics was introduces for a single electron with a spin. One thus must contemplate where do those internal energies originate? The answer to this question seems to come from measurement theory [24,25]. Fisher information is a basic notion of measurement theory, and is a figure of merit of a measurement quality of any quantity. It was shown [25] that this notion is the internal energy of a spin less electron (up to a proportionality constant) and can be used to partially interpret the internal energy of an electron with spin. An attempt to derive most physical theories from Fisher information is due to Frieden [26]. It was suggested [27] that there exists a velocity field such that the Fisher information will give a complete explanation for the spin fluid internal energy. It was also suggested that one may define comoving scalar fields as in ideal fluid mechanics, however, this was only demonstrated implicitly but not explicitly. A common feature of previous work on the fluid and Fisher information interpretation of quantum mechanics, is the negligence of electromagnetic interaction thus setting the vector potential to zero. This was recently corrected in [28].

Ehrenfest [29] published his paper in 1927 as well with the title: “Remark on the approximate validity of classical mechanics within quantum mechanics”. Using this approach we can accept the orthodox Copenhagen’s interpretation denying a trajectory of the electron but at the same time accept the existence of a trajectory of the electron’s position vector expectation value through Ehrenfest theorem. The Ehrenfest approach is thus independent of interpretation, and can be applied according to both the Copenhagen and Bohm schools. However, only in the Bohm approach may one compare the trajectory of the electron to that of its expectation value.

We will begin this paper by reminding the reader of the basic equation describing the motion of a classical electron. This will be followed by a discussion of Schrödinger equation with a non trivial vector potential and its interpretation in terms of Bohmian equation of motion with a quantum force correction. Then we introduce Pauli’s equation with a vector potential and interpret it in terms a Bohmian equation of motion with a quantum force correction which is different from the Schrödinger case. Finally we derive an equation for Pauli’s electron position vector expectation value using Ehrenfest theorem and compare the result to the results obtained in Bohm’s approach, similarities and differences will arise, a concluding section will follow discussing the Stern–Gerlach experiment.

## 2. A Classical Charged Particle

Consider a classical particle with the coordinates $\vec{x}\left(t\right)$, mass *m* and charge *e* interacting with a given electromagnetic vector potential $\vec{A}\left(\vec{x},t\right)$ and scalar potential $\varphi (\vec{x},t)$. We will not be interested in the effects of the particle on the field and thus consider the field as “external”. The action of said particle is:

(1) $$\begin{array}{ccc} A & = & \int_{t1}^{t2}Ldt,\;L=L_{0}+L_{i}\\ L_{0} & \equiv & \frac{1}{2}mv^{2},\;L_{i}\equiv e\left(\vec{A}\cdot \vec{v}-\varphi \right),\;\vec{v}\equiv \frac{d\vec{x}}{dt}\equiv \dot{\vec{x}},\;v=\left|\vec{v}\right|. \end{array}$$

The variation of the two parts of the Lagrangian are given by:

(2) $$\delta L_{0}=m\dot{\vec{x}}\cdot \delta \dot{\vec{x}}=\frac{d(m\vec{v}\cdot \delta \vec{x})}{dt}-m\dot{\vec{v}}\cdot \delta \vec{x}$$

(3) $$\begin{array}{ccc} \delta L_{i} & = & e\left(\delta \vec{A}\cdot \vec{v}+\vec{A}\cdot \delta \dot{\vec{x}}-\delta \varphi \right)\\ & = & e\left(\partial_{k}\vec{A}\cdot \vec{v}\delta x_{k}+\frac{d(\vec{A}\cdot \delta \vec{x})}{dt}-\delta \vec{x}\cdot \frac{d\vec{A}}{dt}-\vec{\nabla }\varphi \cdot \delta \vec{x}\right), \end{array}$$

in the above $\partial_{k}\equiv \frac{\partial }{\partial x_{k}}$ and $\vec{\nabla }\equiv \left(\frac{\partial }{\partial x},\frac{\partial }{\partial y},\frac{\partial }{\partial z}\right)\equiv \left(\frac{\partial }{\partial x_{1}},\frac{\partial }{\partial x_{2}},\frac{\partial }{\partial x_{3}}\right)$. We use the Einstein summation convention in which a Latin index (say k,l) takes one of the values k,l∈[1,2,3]. We may write the total time derivative of $\vec{A}$ as:

(4) $$\frac{d\vec{A}\left(\vec{x}\left(t\right),t\right)}{dt}=\partial_{t}\vec{A}+v_{l}\partial_{l}\vec{A},\;\partial_{t}\equiv \frac{\partial }{\partial t}.$$

Thus, the variation $\delta L_{i}$ can be written in the following form:

(5) $$\delta L_{i}=\frac{d(e\vec{A}\cdot \delta \vec{x})}{dt}+e\left[\left(\partial_{k}A_{l}-\partial_{l}A_{k}\right)v_{l}-\partial_{t}A_{k}-\partial_{k}\varphi \right]\delta x_{k},$$

Defining the electric and magnetic fields in the standard way:

(6) $$\vec{B}\equiv \vec{\nabla }\times \vec{A},\;\vec{E}\equiv -\partial_{t}\vec{A}-\vec{\nabla }\varphi ,$$

it follows that:

(7) $$\epsilon_{kln}B_{n}=\partial_{k}A_{l}-\partial_{l}A_{k},\;E_{k}=-\partial_{t}A_{k}-\partial_{k}\varphi ,$$

in which $\epsilon_{kln}$ is the three-index antisymmetric tensor. Thus, we may write $\delta L_{i}$ as:

(8) $$\delta L_{i}=\frac{d(e\vec{A}\cdot \delta \vec{x})}{dt}+e\left[\epsilon_{kln}v_{l}B_{n}+E_{k}\right]\delta x_{k}=\frac{d(e\vec{A}\cdot \delta \vec{x})}{dt}+e\left[\vec{v}\times \vec{B}+\vec{E}\right]\cdot \delta \vec{x}.$$

We use the standard definition of the Lorentz force (MKS units):

(9) $${\vec{F}}_{L}\equiv e\left[\vec{v}\times \vec{B}+\vec{E}\right]$$

to write:

(10) $$\delta L_{i}=\frac{d(e\vec{A}\cdot \delta \vec{x})}{dt}+{\vec{F}}_{L}\cdot \delta \vec{x}.$$

Combining the variation of $L_{i}$ given in Equation (10) and the variation of $L_{0}$ given in Equation (2), it follows from Equation (1) that the variation of *L* is:

(11) $$\delta L=\delta L_{0}+\delta L_{i}=\frac{d\left(\left(m\vec{v}+e\vec{A}\right)\cdot \delta \vec{x}\right)}{dt}+\left(-m\dot{\vec{v}}+{\vec{F}}_{L}\right)\cdot \delta \vec{x}.$$

Thus, the variation of the action is:

(12) $$\delta A=\int_{t1}^{t2}\delta Ldt={\left.\left(m\vec{v}+e\vec{A}\right)\cdot \delta \vec{x}\right|}_{t1}^{t2}-\int_{t1}^{t2}\left(m\dot{\vec{v}}-{\vec{F}}_{L}\right)\cdot \delta \vec{x}dt.$$

Since the classical trajectory is such that the variation of the action on it vanishes for a small modification of the trajectory $\delta \vec{x}$ that vanishes at t1 and t2 but is otherwise arbitrary it follows that:

(13) $$m\dot{\vec{v}}={\vec{F}}_{L}=e\left[\vec{v}\times \vec{B}+\vec{E}\right]\Rightarrow \dot{\vec{v}}=\frac{e}{m}\left[\vec{v}\times \vec{B}+\vec{E}\right].$$

Thus, the dynamics of a classical particle in a given electric and magnetic field is described by a single number, the ratio between its charge and mass:

(14) $$k\equiv \frac{e}{m}\;\Rightarrow \;\dot{\vec{v}}=k\left[\vec{v}\times \vec{B}+\vec{E}\right].$$

The reader is reminded that the connection between the electromagnetic potentials and the fields is not unique. Indeed performing a gauge transformation to obtain a new set of potentials:

(15) $$\vec{A^{\prime }}=\vec{A}+\vec{\nabla }\Lambda ,\;\varphi^{\prime }=\varphi -\partial_{t}\Lambda .$$

we obtain the same fields:

(16) $$\vec{B^{\prime }}=\vec{\nabla }\times \vec{A^{\prime }}=\vec{\nabla }\times \vec{A}=\vec{B},\;\vec{E^{\prime }}=-\partial_{t}\vec{A^{\prime }}-\vec{\nabla }\varphi^{\prime }=-\partial_{t}\vec{A}-\vec{\nabla }\varphi =\vec{E}.$$

## 3. Schrödinger’s Theory

Quantum mechanics according to the Copenhagen interpretation has lost faith in our ability to predict precisely the whereabouts of even a single particle. What the theory does predict precisely is the evolution in time of a quantity denoted “the quantum wave function”, which is related to a quantum particle whereabouts in a statistical manner. This evolution is described by an equation suggested by Schrödinger [30]:

(17) $$i\hbar \dot{\psi }={\hat{H}}_{S}\psi ,\;{\hat{H}}_{S}=-\frac{1}{2m}{\left(\hbar \vec{\nabla }-ie\vec{A}\right)}^{2}+e\varphi$$

in the above $i=\sqrt{-1}$ and ψ is the complex wave function. $\dot{\psi }=\frac{\partial \psi }{\partial t}$ is the partial time derivative of the wave function. $\hbar =\frac{h}{2\pi }$ is Planck’s constant divided by 2π and *m* is the particles mass. However, this presentation of quantum mechanics is rather abstract and does not give any physical picture regarding the meaning of the quantities involved. Thus we write the quantum wave function using its modulus *a* and phase ϕ:

(18) $$\psi =ae^{i\phi }.$$

We define the velocity field:

(19) $${\vec{v}}_{S}=\frac{\hbar }{m}\vec{\nabla }\phi -\frac{e}{m}\vec{A}$$

and the mass density is defined as:

(20) $$\hat{\rho }=ma^{2}.$$

It is easy to show from Equation (17) that the continuity equation is satisfied:

(21) $$\frac{\partial \hat{\rho }}{\partial t}+\vec{\nabla }\cdot \left(\hat{\rho }{\vec{v}}_{S}\right)=0$$

Hence ${\vec{v}}_{S}$ field is the velocity associated with mass conservation. However, it is also the mass associate with probability $a^{2}$ (by Born’s interpretational postulate) and charge density $\rho =ea^{2}$. The equation for the phase ϕ derived from Equation (17) is as follows:

(22) $$\hbar \frac{\partial \phi }{\partial t}+\frac{1}{2m}{\left(\hbar \vec{\nabla }\phi -e\vec{A}\right)}^{2}+e\varphi =\frac{\hbar^{2}\nabla^{2}a}{2ma}$$

In term of the velocity defined in Equation (19) one obtains the following equation of motion (see Madelung [5] and Holland [3]):

(23) $$\frac{d{\vec{v}}_{S}}{dt}=\frac{\partial {\vec{v}}_{S}}{\partial t}+\left({\vec{v}}_{S}\cdot \vec{\nabla }\right){\vec{v}}_{S}=-\vec{\nabla }\frac{Q}{m}+k\left(\vec{E}+\vec{v}\times \vec{B}\right)$$

The right hand side of the above equation contains the “quantum correction”:

(24) $$Q=-\frac{\hbar^{2}}{2m}\frac{{\vec{\nabla }}^{2}\sqrt{\hat{\rho }}}{\sqrt{\hat{\rho }}}.$$

For the meaning of this correction in terms of information theory see: [23,25,27]. These results illustrate the advantages of using the two variables, phase and modulus, to obtain equations of motion that have a substantially different form than the familiar Schrödinger equation (although having the same mathematical content) and have straightforward physical interpretations [2].

The quantum correction *Q* will of course disappear in the classical limit ℏ→0, but even if one intends to consider the quantum equation in its full rigor, one needs to take into account the expansion of an unconfined wave function. As *Q* is related to the typical gradient of the wave function amplitude it follows that as the function becomes smeared over time and the gradient becomes small the quantum correction becomes negligible. To put in quantitative terms:

(25) $${\vec{F}}_{Q}=-\vec{\nabla }Q≃\frac{\hbar^{2}}{2mL_{R}^{3}},\;L_{R}≃\frac{R}{|\vec{\nabla }R|}$$

in which $L_{R}$ is the typical length of the amplitudes gradient. Thus:

(26) $$|F_{Q}\left|<<\right|F_{L}|\Rightarrow L_{R}>>L_{Rc}={\left(\frac{\hbar^{2}}{2mF_{L}}\right)}^{\frac{1}{3}}.$$

in which ${\vec{F}}_{L}$ is the classical Lorentz force given in Equation (9). If an electron transverses a macroscopic length this terms seems unimportant.

## 4. Pauli’s Theory

Schrödinger’s quantum mechanics is limited to the description of spin less particles. Indeed the need for spin became necessary as Schrödinger equation could not account for the result of the Stern–Gerlach experiments, predicting a single spot instead of the two spots obtained for hydrogen atoms. Thus Pauli introduced his equation for a non-relativistic particle with spin, given by:

(27) $$i\hbar \dot{\psi }={\hat{H}}_{P}\psi ,\;{\hat{H}}_{P}=-\frac{\hbar^{2}}{2m}{\left[\vec{\nabla }-\frac{ie}{\hbar }\vec{A}\right]}^{2}+\mu \vec{B}\cdot \vec{\sigma }+e\varphi ={\hat{H}}_{S}\;I+\mu \vec{B}\cdot \vec{\sigma }$$

ψ here is a two dimensional complex column vector (also denoted as spinor), ${\hat{H}}_{P}$ is a two dimensional Hermitian operator matrix, μ is the magnetic moment of the particle, and *I* is a two dimensional unit matrix. $\vec{\sigma }$ is a vector of two dimensional Pauli matrices which can be represented as follows:

(28) $$\sigma_{1}=\left(\begin{array}{cc} 0 & 1\\ 1 & 0 \end{array}\right),\;\sigma_{2}=\left(\begin{array}{cc} 0 & -i\\ i & 0 \end{array}\right),\;\sigma_{3}=\left(\begin{array}{cc} 1 & 0\\ 0 & -1 \end{array}\right).$$

The ad hoc nature of this equation was later amended as it became clear that this is the non relativistic limit of the relativistic Dirac equation. A spinor ψ satisfying Equation (27) must also satisfy a continuity equation of the form:

(29) $$\frac{\partial \rho_{p}}{\partial t}+\vec{\nabla }\cdot \vec{j}=0.$$

In the above:

(30) $$\rho_{p}=\psi^{†}\psi ,\;\vec{j}=\frac{\hbar }{2mi}\left[\psi^{†}\vec{\nabla }\psi -\left(\vec{\nabla }\psi^{†}\right)\psi \right]-k\vec{A}\rho_{p}.$$

The symbol $\psi^{†}$ represents a row spinor (the transpose) whose components are equal to the complex conjugate of the column spinor ψ. Comparing the standard continuity equation to Equation (29) suggests the definition of a velocity field as follows [3]:

(31) $$\vec{v}=\frac{\vec{j}}{\rho_{p}}=\frac{\hbar }{2mi\rho_{p}}\left[\psi^{†}\vec{\nabla }\psi -\left(\vec{\nabla }\psi^{†}\right)\psi \right]-k\vec{A}.$$

Holland [3] has suggested the following representation of the spinor:

(32) $$\psi =Re^{i\frac{\chi }{2}}\left(\begin{array}{c} \cos\left(\frac{\theta }{2}\right)e^{i\frac{\phi }{2}}\\ i\sin\left(\frac{\theta }{2}\right)e^{-i\frac{\phi }{2}} \end{array}\right)\equiv \left(\begin{array}{c} \psi_{↑}\\ \psi_{↓} \end{array}\right).$$

In terms of this representation the density is given as:

(33) $$R^{2}=\psi^{†}\psi =\rho_{p}\Rightarrow R=\sqrt{\rho_{p}}.$$

The mass density is given as:

(34) $$\hat{\rho }=m\psi^{†}\psi =mR^{2}=m\rho_{p}.$$

The probability amplitudes for spin up and spin down electrons are given by:

(35) $$a_{↑}=\left|\psi_{↑}\right|=R\left|\cos\frac{\theta }{2}\right|,\;a_{↓}=\left|\psi_{↓}\right|=R\left|\sin\frac{\theta }{2}\right|$$

Let us now look at the expectation value of the spin:

(36) $$<\frac{\hbar }{2}\vec{\sigma }>=\frac{\hbar }{2}\int \psi^{†}\vec{\sigma }\psi d^{3}x=\frac{\hbar }{2}\int \left(\frac{\psi^{†}\vec{\sigma }\psi }{\rho_{p}}\right)\rho_{p}d^{3}x$$

The spin density can be calculated using the representation given in Equation (32) as:

(37) $$\hat{s}\equiv \frac{\psi^{†}\vec{\sigma }\psi }{\rho_{p}}=\left(\sin\theta \sin\phi ,\sin\theta \cos\phi ,\cos\theta \right),\;\left|\hat{s}\right|=\sqrt{\hat{s}\cdot \hat{s}}=1.$$

This gives an easy physical interpretation to the variables θ,ϕ as angles which describe the projection of the spin density on the axes. θ is the elevation angle of the spin density vector and ϕ is the azimuthal angle of the same. The velocity field can now be calculated by inserting ψ given in Equation (32) into Equation (31):

(38) $$\vec{v}=\frac{\hbar }{2m}\left(\vec{\nabla }\chi +\cos\theta \vec{\nabla }\phi \right)-k\vec{A}.$$

We are now in a position to calculate the material derivative of the velocity and obtain the equation of motion for a particle with ([3], p. 393, Equation (9.3.19)):

(39) $$\frac{d\vec{v}}{dt}=-\vec{\nabla }\left(\frac{Q}{m}\right)-{\left(\frac{\hbar }{2m}\right)}^{2}\frac{1}{\rho_{p}}\partial_{k}\left(\rho_{p}\vec{\nabla }{\hat{s}}_{j}\partial_{k}{\hat{s}}_{j}\right)+k\left(\vec{E}+\vec{v}\times \vec{B}\right)-\frac{\mu }{m}\left(\vec{\nabla }B_{j}\right){\hat{s}}_{j}.$$

The Pauli equation of motion differs from the classical equation motion and the Schrödinger equation of motion. In addition to the Schrödinger quantum force correction we have an additional spin quantum force correction:

(40) $${\vec{F}}_{QS}\equiv -\frac{\hbar^{2}}{4m}\frac{1}{\rho_{p}}\partial_{k}\left(\rho_{p}\vec{\nabla }{\hat{s}}_{j}\partial_{k}{\hat{s}}_{j}\right)=-\frac{\hbar^{2}}{4m}\left[\partial_{k}\left(\vec{\nabla }{\hat{s}}_{j}\partial_{k}{\hat{s}}_{j}\right)+\frac{\partial_{k}\rho_{p}}{\rho_{p}}\vec{\nabla }{\hat{s}}_{j}\partial_{k}{\hat{s}}_{j}\right]$$

as well as a term characterizing the interaction of the spin with a gradient of the magnetic field.

(41) $${\vec{F}}_{gradBS}\equiv -\mu \left(\vec{\nabla }B_{j}\right)s_{j}$$

As both the upper and lower spin components of the wave function are expanding in free space the gradients which appear in ${\vec{F}}_{QS}$ will tend to diminish for any macroscopic scale making this force negligible. To estimate the condition qualitatively we introduce the typical spin length:

(42) $$L_{s}=\min_{\;i\in \{1,2,3\}}\;{\left|\vec{\nabla }{\hat{s}}_{i}\right|}^{-1}$$

Using the above definition we may estimate the spin quantum force:

(43) $$F_{QS}\approx \frac{\hbar^{2}}{4m}\left[\frac{1}{L_{s}^{3}}+\frac{1}{L_{s}^{2}L_{R}}\right]=\frac{\hbar^{2}}{4mL_{s}^{2}}\left[\frac{1}{L_{s}}+\frac{1}{L_{R}}\right]$$

this suggested the definition of the hybrid typical length:

(44) $$L_{sR}={\left[\frac{1}{L_{s}}+\frac{1}{L_{R}}\right]}^{-1}=\left\{\begin{array}{cc} L_{s} & L_{s}\ll L_{R}\\ L_{R} & L_{R}\ll L_{s} \end{array}\right..$$

In terms of this typical length we may write:

(45) $$F_{QS}\approx \frac{\hbar^{2}}{4mL_{s}^{2}L_{sR}}$$

Thus the conditions for a classical trajectory become:

(46) $$F_{QS}\ll F_{L}\Rightarrow L_{s}^{2}L_{sR}\gg \frac{\hbar^{2}}{4mF}\Rightarrow L_{s}\gg \left\{\begin{array}{cc} {\left(\frac{\hbar^{2}}{4mF_{L}}\right)}^{\frac{1}{3}} & L_{s}\ll L_{R}\\ {\left(\frac{\hbar^{2}}{4mF_{L}L_{R}}\right)}^{\frac{1}{2}} & L_{R}\ll L_{s} \end{array}\right..$$

Another important equation derived from Equation (27) is the equation of motion for the spin orientation vector ([3], p. 392, Equation (9.3.16)):

(47) $$\frac{d\hat{s}}{dt}=\frac{2\mu }{\hbar }{\vec{B}}_{eff}\times \hat{s},\;{\vec{B}}_{eff}=\vec{B}-\frac{\hbar^{2}}{4\mu mR^{2}}\partial_{i}\left(\rho \partial_{i}\hat{s}\right)$$

The quantum correction to the magnetic field explains [3] why a spin picks up the orientation of the field in a Stern–Gerlach experiment instead of precessing around it as a classical magnetic dipole would.

Finally we remark that despite the fact that the electron is (as far as the empirical evidence suggests) a point particle and thus cannot rotate with respect to its center of mass as a rigid finite body would, there is a long and respectable tradition of attributing to the electron a “classical” spin [31,32,33,34], as if it was rigid body. Despite some success of this approach we regard it as highly non-intuitive.

## 5. Ehrenfest Theorem

According to the Copenhagen school of quantum mechanics, no attribute can be given to the electron unless it can be measured (see for example [35]). Now according to the Heisenberg uncertainty rule, one cannot measure both the position and momentum (which are complementary attributes and do not commute) of the electron at the same time. Hence, two attributes that are needed to define a trajectory: position and direction of propagation cannot be attributed to the electron simultaneously. Thus the above electron equations of motion are not accepted by all quantum physicists. In fact, physicists who follow the Copenhagen school of quantum mechanics declare that a quantum electron does not have a trajectory.

Of course, not all quantum physicists follow the Copenhagen school, as many follow Bohm’s [2] school of thought (Einstein, Holland [3], Durr & Teufel [4] etc.) which do assign a trajectory to the electron, despite the fact that velocity and position cannot be measured at the same time. According to this school of thought a trajectory is an ontological property of the electron and it exists regardless of our ability to measure it (in general it is believed that reality exists regardless of our ability to observe it). The reader is also referred to [36] which study proton trajectories. Those differ from the subject of the current paper which is electrons. Protons are not point particles like electrons and thus they can “spin” also in a classical sense.

However, all quantum physicists agree that one can describe the trajectory of the expectation value of various operators such as position and momentum associated with the electron’s trajectory. This calculation is carried out through the Ehrenfest Theorem [37]. The theorem states that for every quantum operator $A_{o}$ with expectation value:

(48) $$<A_{o}>=\int d^{3}x\psi^{†}A_{o}\psi$$

the following equality holds:

(49) $$\frac{d<A_{o}>}{dt}=<\frac{\partial A_{o}}{\partial t}>+\frac{1}{i\hbar }<\left[A_{o},\hat{H}\right]>,\;\left[A_{o},\hat{H}\right]\equiv A_{o}\hat{H}-\hat{H}A_{o}.$$

The position and velocity operators defined as [37]:

(50) $${\vec{x}}_{o}\equiv \vec{x},\;{\vec{v}}_{o}\equiv \frac{1}{m}\left({\vec{p}}_{o}-e\vec{A}\right)=\frac{1}{m}\left(-i\hbar \vec{\nabla }-e\vec{A}\right)$$

For a Schrödinger’s electron (that is without spin) the following results are obtained by Griffiths [37] by inserting the above operators into Equation (49):

(51) $$\begin{array}{ccc} \frac{d<{\vec{x}}_{o}>}{dt} & = & \frac{1}{i\hbar }<\left[\vec{x},{\hat{H}}_{S}\right]>=<{\vec{v}}_{o}>\\ \frac{d<{\vec{v}}_{o}>}{dt} & = & <\frac{\partial {\vec{v}}_{o}}{\partial t}>+\frac{1}{i\hbar }<\left[{\vec{v}}_{o},{\hat{H}}_{S}\right]>\\ & = & \frac{k}{2}<{\vec{v}}_{o}\times \vec{B}-\vec{B}\times {\vec{v}}_{o}>+k<\vec{E}> \end{array}$$

Thus the electron position expectation value satisfies the equation:

(52) $$\frac{d^{2}<{\vec{x}}_{o}>}{dt^{2}}=\frac{k}{2}<{\vec{v}}_{o}\times \vec{B}-\vec{B}\times {\vec{v}}_{o}>+k<\vec{E}>$$

this equation resembles the classical and quantum equations of motion but also differs from them in many important aspects. First let us compare it with Equation (13), let us also assume that $\vec{B}=0$. In this case:

(53) $$\frac{d^{2}<\vec{x}>}{dt^{2}}=k<\vec{E}\left(\vec{x},t\right)>\neq k\vec{E}\left(<\vec{x}>,t\right),$$

thus as noted by many authors, even in this case the expectation value equations differ from the classical equation of motion except for a very restrictive class of linear electric fields. The difference is even more pronounced for the case $\vec{B}\neq 0$ which only takes a conventional “Lorentz force” form for a constant magnetic field $\vec{B}$ [37]:

(54) $$\frac{d^{2}<\vec{x}>}{dt^{2}}=k\left(<{\vec{v}}_{o}>\times \vec{B}+<\vec{E}>\right).$$

Comparing Equation (52) with the quantum motion Equation (23), we see that the expectation value equation does not contain a quantum force term, which is a further justification to our assumption that this term may be neglected on macroscopic scales.

Let us now turn our attention to the more realistic Pauli electron which does possess spin, Equation (51) now take the form:

(55) $$\begin{array}{ccc} & & \frac{d<{\vec{x}}_{o}>}{dt}=\frac{1}{i\hbar }<\left[\vec{x},{\hat{H}}_{P}\right]>=\frac{1}{i\hbar }<\left[\vec{x},{\hat{H}}_{S}\right]>=<{\vec{v}}_{o}>\\ & & \frac{d<{\vec{v}}_{o}>}{dt}=<\frac{\partial {\vec{v}}_{o}}{\partial t}>+\frac{1}{i\hbar }<\left[{\vec{v}}_{o},{\hat{H}}_{P}\right]>\\ & & =<\frac{\partial {\vec{v}}_{o}}{\partial t}>+\frac{1}{i\hbar }<\left[{\vec{v}}_{o},{\hat{H}}_{S}\right]>+\frac{1}{i\hbar }<\left[{\vec{v}}_{o},\mu \vec{B}\cdot \vec{\sigma }\right]>\\ & & =\frac{k}{2}<{\vec{v}}_{o}\times \vec{B}-\vec{B}\times {\vec{v}}_{o}>+k<\vec{E}>+\frac{\mu }{i\hbar }<\left[{\vec{v}}_{o},B_{i}\right]\sigma_{i}>, \end{array}$$

in which in the last term we use the Einstein summation convention. However:

(56) $$\left[{\vec{v}}_{o},B_{i}\right]=\frac{1}{m}\left[-i\hbar \vec{\nabla }-e\vec{A},B_{i}\right]=-\frac{i\hbar }{m}\left[\vec{\nabla },B_{i}\right]$$

The value of this commutation relation can be deduced by operating with the above operator on an arbitrary wave function ψ.

(57) $$\left[{\vec{v}}_{o},B_{i}\right]\psi =-\frac{i\hbar }{m}\left[\vec{\nabla },B_{i}\right]\psi =-\frac{i\hbar }{m}\left(\vec{\nabla }\left(B_{i}\psi \right)-B_{i}\vec{\nabla }\psi \right)=-\frac{i\hbar }{m}\left(\vec{\nabla }B_{i}\right)\psi$$

Or in operator jargon:

(58) $$\left[{\vec{v}}_{o},B_{i}\right]=-\frac{i\hbar }{m}\left(\vec{\nabla }B_{i}\right)$$

It thus follows that:

(59) $$\frac{\mu }{i\hbar }<\left[{\vec{v}}_{o},B_{i}\right]\sigma_{i}>=-\frac{\mu }{m}<\left(\vec{\nabla }B_{i}\right)\sigma_{i}>=-\frac{\mu }{m}<\left(\vec{\nabla }B_{i}\right){\hat{s}}_{i}>$$

in which we have used Equation (37). Inserting Equation (59) into Equation (55):

(60) $$\frac{d^{2}<{\vec{x}}_{o}>}{dt^{2}}=\frac{d<{\vec{v}}_{o}>}{dt}=\frac{k}{2}<{\vec{v}}_{o}\times \vec{B}-\vec{B}\times {\vec{v}}_{o}>+k<\vec{E}>-\frac{\mu }{m}<{\hat{s}}_{i}\vec{\nabla }B_{i}>$$

comparing the above equation to Equation (39) it follows that the only quantum force surviving the expectation value averaging is the one describing the effect of the magnetic field gradient on the spin vector which is in accordance with what should be expected in the macroscopic limit thus leading to the Stern–Gerlach experiment.

## 6. Conclusions: Spin Orientation and the Stern–Gerlach Experiment

We have seen how the Ehrenfest theorem approach causes “quantum forces” to disappear. Those forces disappear also in Bohm’s approach if one consider macroscopic scales of propagation.

**Figure 1:**
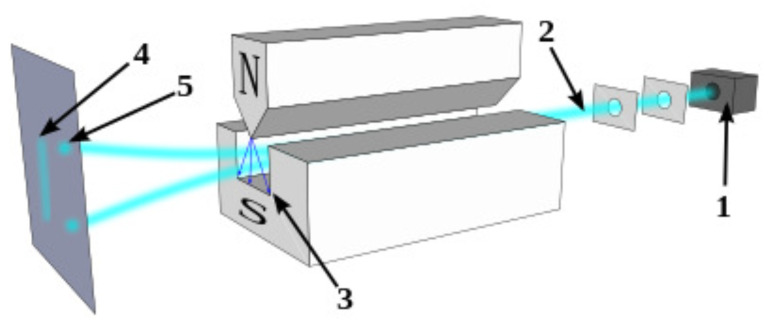
A schematics of Stern–Gerlach experiment. Neutral particles travelling through an inhomogeneous magnetic field, and being deflected up or down depending on their spin; (1) particle source, (2) beam of particles, (3) inhomogeneous magnetic field, (4) classically expected result (neglecting the quantum spin force), (5) observed result.

The Stern–Gerlach experiment is an example of using the spin force term given in Equation (41) to separate spin up and spin down particles thus obtaining from a single ray of particles two spots. If the magnetic field gradient is dominant in a single direction (say *z*) we may write Equation (41) as:

(61) $$F_{gradBS\;z}=-\mu \left(\partial_{z}B_{j}\right){\hat{s}}_{j}$$

hence depending on the values ${\hat{s}}_{j}$ some particle will move up and some will move down creating two spots (see Figure 1).

The Stern–Gerlach experiment is usually performed with a neutral particle, not with charged particles such as electrons. The reason for this is that generally speaking the classical Lorentz forces are much stronger than the quantum spin force and thus the two-spot effect is not observed. Holland shows by simulating Equation (47) that the spins in a Stern–Gerlach rotate in the direction or opposite to the direction of the magnetic field depending on the trajectory of the particle, that is to which spot it belongs (see Holland [3] Figure 9.13). Notice, however, that from an energy perspective the lowest energy belong to the case in which the spin (and thus its related magnetic dipole) point at the direction of the field.

The energy value is given by the expectation value of the Hamiltonian:

(62) $$E=<{\hat{H}}_{P}>=<{\hat{H}}_{S}>+\mu <\vec{B}\cdot \vec{\sigma }>.$$

If the direction of the field $\vec{B}$ is defined as a *z* direction it follows that:

(63) $$E=<{\hat{H}}_{S}>+\mu <B_{z}\sigma_{z}>=<{\hat{H}}_{S}>+\mu \int d^{3}xB_{z}\left(a_{↑}^{2}-a_{↓}^{2}\right).$$

So particles with a definite spin direction (up or down) may have an upper or lower energy depending on the value of μ. For an electron μ is the Bohr magneton:

(64) $$\mu =\mu_{B}=\frac{|e|\hbar }{2m}.$$

and thus lower spin electrons will have a lower energy, if $B_{z}$ is constant we may write this term in the “classical” form using a magnetic dipole:

(65) $$E=<{\hat{H}}_{S}>-\vec{\mu }\cdot \vec{B},\;\vec{\mu }=-\mu \int d^{3}x\left(a_{↑}^{2}-a_{↓}^{2}\right)\hat{z}=\mu \int d^{3}x\left(a_{↓}^{2}-a_{↑}^{2}\right)\hat{z}.$$

hence the magnetic dipole will point in the direction of the field for a lower energy configurations (spin down) and in the opposite direction for the higher energy configuration (spin up). As systems tend to relax to their lower energy state, one may ask why do the particles in a Stern–Gerlach experiment do not relax to the spin down configuration and instead split to beams of spin up and spin down with about the same size? The answer may be connected to the fact that in this type of experiment the electrons feel the magnetic field for only a short while and do not have enough time to relax to their minimum energy configurations.

This is not the case in NMR and MRI experiment in which the magnetic dipoles are under the influence of a strong magnetic field, for a long duration. In those cases the magnetization defined as:

(66) $$\vec{M}=\rho_{P}\vec{\mu }$$

satisfies the Bloch phenomenological equations:

(67) $$\begin{array}{ccc} \frac{dM_{x}}{dt} & = & \gamma_{gyro}{\left(\vec{M}\times \vec{B}\right)}_{x}-\frac{M_{x}}{T_{2}}\\ \frac{dM_{y}}{dt} & = & \gamma_{gyro}{\left(\vec{M}\times \vec{B}\right)}_{y}-\frac{M_{y}}{T_{2}}\\ \frac{dM_{z}}{dt} & = & \gamma_{gyro}{\left(\vec{M}\times \vec{B}\right)}_{z}-\frac{M_{z}-M_{0}}{T_{1}} \end{array}$$

in the above $T_{1}$ and $T_{2}$ are typical relaxation times and γ is a gyromagnetic ratio which for an electron takes the value:

(68) $$\gamma_{gyro}=\frac{2\mu_{B}}{\hbar }$$

**Figure 2:**
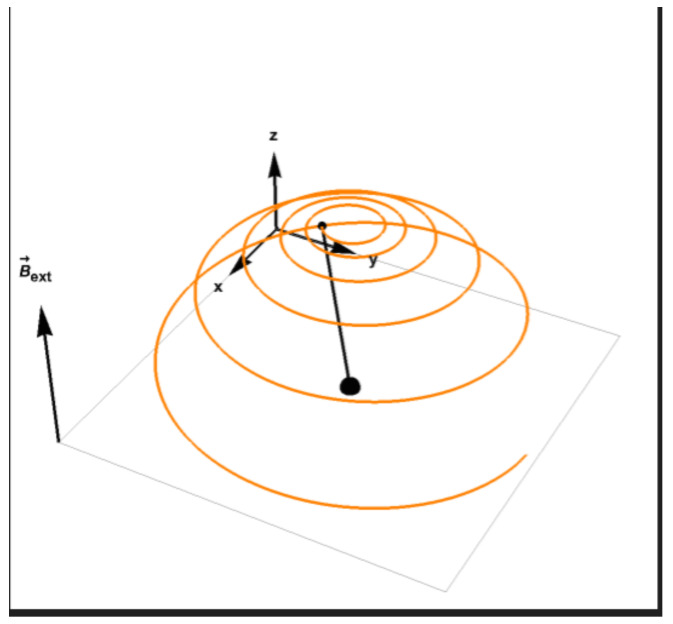
The evolution of magnetization towards relaxation, the tip of the magnetization vector is described by the orange line.

The magnetization satisfying the above equation eventually relaxes to the direction of the field which is the minimal energy configuration (Figure 2).

As a future direction to the current research it may be interesting to study the same problem for a fully relativistic electron, however, this will require using a Dirac equation rather than Pauli’s equation.

## Data Availability

Not applicable.
